# “Chronic myelogenous leukemia in primary blast crisis” rather than “de novo *BCR‐ABL1*‐positive acute myeloid leukemia”

**DOI:** 10.1002/ccr3.937

**Published:** 2017-04-04

**Authors:** Cedric Pastoret, Roch Houot

**Affiliations:** ^1^ Laboratory of HematologyUniversity HospitalRennesFrance; ^2^ Department of Clinical HematologyUniversity HospitalRennesFrance

**Keywords:** Basophiloblast, *BCR‐ABL1*‐positive acute myeloid leukemia, chronic myeloid leukemia

## Abstract

Differentiating chronic myelogenous leukemia in primary blast crisis (CML‐BC) from de novo *BCR‐ABL1*‐positive acute myeloid leukemia (AML) is a diagnostic challenge with therapeutic consequences. In our case, a basophilia, the presence of the Philadelphia chromosome in all metaphases and the strict exclusion of molecular hallmarks of AML lead us to retain the diagnosis of CML‐BC rather than *BCR‐ABL1+* AML.

## Introduction

De novo *BCR‐ABL1*‐positive AML is a rare disease recently recognized as a provisional entity in the 2016 revision of the World Health Organization (WHO) classification of myeloid neoplasms 1. However, the diagnosis of chronic myelogenous leukemia in primary blast crisis (CML‐BC) has to be systematically considered, even in the absence of preceding leukocytosis or splenomegaly. Indeed, patients with CML‐BC may benefit from a tyrosine kinase inhibitor rather than AML chemotherapy regimen 2, 3.

## Case Report

**Figure 1:**
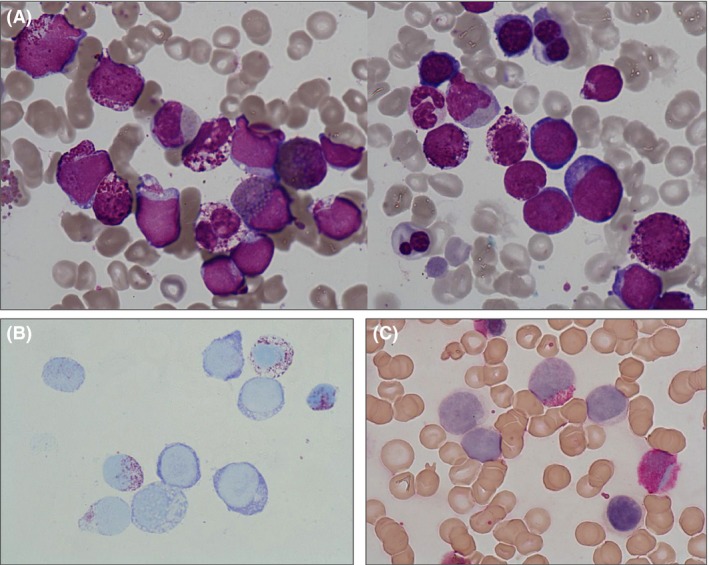
(A) Bone marrow aspirate smear showing numerous basophiloblasts, abnormal mature basophils with dispersed granules, eosinophils, and signs of myelodysplasia on erythroid lineage such as nuclear abnormalities and deshemoglobinization (May–Grunwald–Giemsa stain, × 1000). (B) Positivity of basophiloblasts and mature basophils on the cytochemical stain with toluidine blue. (C) Positivity of one blast on the stain for myeloperoxidase (alpha‐naphthol pyronine as substrate).

A 63‐year‐old woman presented to the emergency department with asthenia, persistent cough, expectorations, and fever despite antibiotics for 5 days. She did not report previous history of hematologic malignancies. Physical examination found no splenomegaly. Complete blood count revealed an anemia (hemoglobin 9.8 g/dL), a thrombocytopenia (73 × 10^9^/L), and a leukocytosis (42.2 × 10^9^/L) with a basophilia (8.4 × 10^9^/L), 39% of blasts, and 3% of myelocytes. Bone marrow studies showed a hypercellular marrow with 38% of blasts with deep purple granules in the May–Grunwald–Giemsa stain (Fig. 1A). These blasts, as well as the mature basophils, were metachromatic in the toluidine blue stain and were described as basophilobasts (Fig. 1B). Only a few blasts showed MPO positivity (Fig. 1C). Mature basophils accounted for 15% of all nucleated cells and often exhibited atypical dispersed granules. There was a slight increase in eosinophils. Moreover, we evidenced important signs of erythroid dysplasia (Fig. 1A). Flow cytometry analysis showed that the blasts expressed bright CD33, CD117, CD13 and were negative for MPO, CD34, HLA‐DR, and B or T‐lymphoid markers, confirming the diagnosis of acute myeloid leukemia (AML). At this stage, the diagnosis of AML with myelodysplasia‐related changes was retained. However, the cytological pattern of acute basophilic leukemia is strongly associated with AML with recurrent cytogenetic abnormalities as t(6;9) and t(9;22), requiring further molecular investigations 4.

Cytogenetic analysis identified the Philadelphia chromosome in 100% of analyzed metaphases with additional chromosomic abnormalities: 46,XX,add(7)(q35),t(9;22)(q34;q11)[5],47,idem,+8[5]. Molecular analysis revealed a p210 *BCR‐ABL1* transcript without mutations in the ABL1‐kinase domain. The detection of this recurrent genenetic abnormality according WHO 2016 classification excludes the diagnostic of AML with myelodysplasia‐related change. Thus, the diagnostics of de novo *BCR‐ABL1*‐positive AML or CML in primary blast crisis have to be discussed. The High‐Throughput Sequencing (MiSeq, Illumina [San Diego, CA]) did not evidence any mutations in genes classically associated with de novo AML or myelodysplastic syndromes (*i.e., NPM1, FLT3, CEPBa, DNMT3A, IDH1, IDH2, TET2, ASXL1, SRSF2, RUNX1, KIT, SF3B1, EZH2, TP53*). No deletion in *IKZF1* or *CNKN2A/2B* genes was evidenced with Multiplex Ligation Probe Assay (SALSA MLPA kit, MRC‐Holland [Amsterdam, the Netherlands]). According to the cytological and molecular pattern of the disease, we finally retained the diagnosis of CML in primary blast crisis rather than de novo *BCR‐ABL1*+ AML.

The patient received an induction therapy with idarubicin, cytarabine and lomustine in combination with a second‐generation tyrosine kinase inhibitor dasatinib (140 mg/day). Bone marrow evaluation at day 30 evidenced a partial response (9% of blasts) according to recent ELN2017 guidelines 5. During the aplasia, she was managed in the intensive care unit for a severe pneumonia with septic choc and digestive hemorrhage. These complications lead us to propose an alternative treatment with 5‐azacytidine (75 mg/m^2^/day SC, 7 days a month) in combination with dasatinib. After 3 months of treatment, the patient reached a complete cytogenetic response. The *BCR‐ABL1* transcript level remained stable at 0.25% IS with a follow‐up of 9 month since the initial presentation. The tolerance was acceptable, and the complete blood count showed a hemoglobin level at 11.8 g/dL, 222 × 10^9^/L platelets, and 2.2 × 10^9^/L neutrophils. The opportunity of allogenic stem cells transplant is actually discussed.

## Discussion

Blastic crisis remains the main therapeutic challenge in CML, even in the tyrosine kinase inhibitors area. Usually, blast crisis arises in patients with known CML, but it can be the initial presentation of the disease. In this case, the distinction between CML‐BC and de novo *BCR‐ABL1*+ AML is a diagnostic dilemma, particularly if in the absence of previous history of leukocytosis or splenomegaly. Neuendorff et al. recently reported an algorithm based on cytological and molecular features 6.

Cytological examination can provide a diagnostic orientation and guide the molecular work‐up. Indeed, the increase in basophils is strongly associated with the diagnosis of CML 6. More than 20% of basophils are sufficient to classify the CML in accelerate phase according to both WHO‐2016 and European LeukemiaNet (ELN‐2013) classifications 1, 7. Basophilia confers a poor prognosis factor integrated in the risk categorization scores EUTOS in CML. Conversely, basophilia is a very rare condition in de novo *BCR‐ABL1* leukemias. In our case, the bone marrow blasts also showed basophilic granules in their cytoplasm. This pattern of acute basophilic leukemia is very rare. It has been reported in association with t(6;9), 12p rearrangements or t(9;22), the latter consisting in the blastic phase of CML in the literature 4. Thus, in our report, cytological examination is in favor of the diagnosis of CML‐BC rather than de novo *BCR‐ABL1* AML. On the other hand, myelodysplastic changes are most often associated with de novo *BCR‐ABL1* AML 6. However, in our case, the absence of cytogenetics abnormalities or mutations associated with myelodysplastic syndrome leads us to exclude the diagnosis of AML with myelodysplasia‐related changes. This case shows the interest of cytological examination at diagnosis to guide the molecular work‐up for AML classification.

The main genetic differences between CML‐BC and *BCR‐ABL1*+ AML can be explained by their distinct physiopathology. Indeed, BCR‐ABL1 is the main driver event in CML, sufficient to initiate the disease. Conversely, applying the two‐hit leukemogenesis model, BCR‐ABL1 consists in a secondary event in AML conferring a proliferative advantage to the cell (class I mutations). It requires an initiating event affecting the myeloid differentiation (class II mutations) to produce the AML phenotype. Consequently, the presence of the Philadelphia chromosome in less than 100% of metaphases during karyotype is the major criterion for the diagnosis of *BCR‐ABL1*+ AML 8, 9. In our case, the presence of the Philadelphia chromosome in all the metaphases, the detection of p210 rather than p190 transcript, and the absence of AML driver mutations were all in favor of the diagnosis of CML‐BC 6, 8, 10. Moreover, trisomy 8 is the most frequent additional cytogenetic alterations observed during the evolution of CML, although it is uncommon in AML 8. Finally, *BCR‐ABL1*+ AML displays characteristics of lymphoid malignancies as blasts frequently express lymphoid markers and harbor deletions of *IKZF1* or *CDKN2A/2B* genes 11. These aberrations are absent in CML‐BC adding a helpful tool in difficult cases. In conclusion, in our case, phenotypic, cytogenetic, and molecular features strongly supported the diagnosis of CML‐BC rather than *BCR‐ABL1*+ AML.

Patients with CML in primary blast crisis should be treated with a tyrosine kinase inhibitor with or without chemotherapy, with the goal of obtaining the best response and proceeding to allogenic stem cell transplantation as quickly as possible 2. In our case, in the absence of a preceding history of chronic myeloid leukemia and organomegaly, the diagnosis of de novo *BCR‐ABL1*+ AML was initially proposed 10. Consequently, an induction chemotherapy was initiated in combination with a second‐generation tyrosine kinase inhibitor as recently recommended by the ELN 5. This treatment failed to reach a complete response and was poorly tolerated. The revision of the initial diagnosis to CML‐BC, the presence of myelodysplastic changes, and the recent experience of Ghez et al. justified the use of dasatinib in combination with 5‐azacytidine 12. Even in the tyrosine kinase inhibitor era, the prognostic of patients with CML‐BC remains poor with few patients surviving longer than 6 months. In our patient, this combination enabled a cytogenetic response within three‐first months and a substantial clinical improvement. This patient is still alive and continues to receive 5‐ azacytidine + dasatinib almost a year after initial diagnosis. This combination may be an interesting alternative for the treatment of CML‐BC ineligible for intensive chemotherapy.

## Conclusion

The diagnosis of CML in primary blast crisis is rare but needs to be systematically excluded before retaining the diagnosis of de novo *BCR‐ABL1*‐positive AML. In absence of splenomegaly or previous history of leukocytosis, it requires a careful cytological examination and extensive molecular investigations.

## Authorship

CP: analyzed the cytological and molecular data and wrote the manuscript and subsequent revisions; RH: manage the patient and critically reviewed the manuscript. All authors interpreted the data and take the responsibility for the accuracy of the case presentation.

## Conflict of Interest

No conflict of interest to report.
